# Three‐Day Bi‐Hemispheric tDCS With Motor Training Shows No Improvement in Pianist's With Musician's Dystonia: A Pilot Study

**DOI:** 10.1111/nyas.70375

**Published:** 2026-08-20

**Authors:** Isabella Ampomah, Michael Großbach, Andre Lee, Eckart Altenmüller

**Affiliations:** ^1^ Hochschule für Musik, Theater und Medien Hannover Germany; ^2^ Department of Neurology, School of Medicine and Health Sciences Carl Von Ossietzky University Oldenburg Oldenburg Germany

**Keywords:** fine‐motor control, focal dystonia, musician's dystonia, transcranial direct current stimulation, treatment of dystonia

## Abstract

Focal hand dystonia in musicians is a task‐specific movement disorder that can impair professional performance, and effective rehabilitative treatments remain limited. Prior work suggested that bi‐hemispheric transcranial direct current stimulation (tDCS) paired with bimanual mirror‐movement training may transiently improve fine motor control in affected pianists. This case‐control pilot study tested whether extending this approach to three consecutive days would produce detectable and sustained benefit. Ten professional pianists with unilateral musician's dystonia and 10 matched healthy pianists received 30 min of bi‐hemispheric tDCS over sensorimotor cortices while performing bimanual mirrored finger exercises on three consecutive days, with follow‐up assessment 10 days later. After exclusions, analyses included eight patients and eight controls for Musical Instrument Digital Interface‐based motor outcomes and six patients and eight controls for video ratings. As anticipated, dystonia patients showed poorer temporal motor control and lower subjective performance ratings than controls. However, the protocol did not produce significant group‐level improvement in objective motor performance, expert‐rated movement impairment or regularity, or subjective performance, nor was benefit retained at 10 days. Video ratings of movement impairment showed acceptable reliability, whereas regularity ratings did not. These findings do not support detectable efficacy of this consecutive‐day protocol under the present conditions and highlight the need for larger randomized, sham‐controlled studies.

## Graphical abstract

Bi‐hemispheric transcranial direct current stimulation (tDCS) paired with bimanual mirror‐movement training may transiently improve fine motor control in pianists affected with focal hand dystonia in musicians, which is a task‐specific movement disorder. This case‐control pilot study tested whether extending this approach to three consecutive days would produce detectable and sustained benefit.

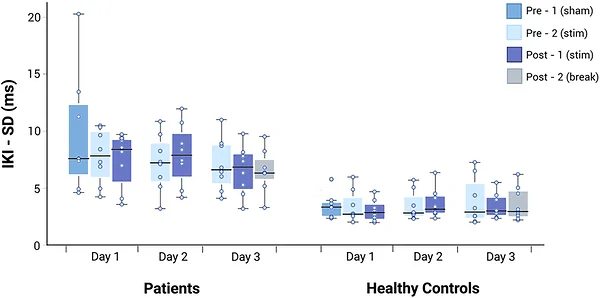

## 1 Introduction

Musician's dystonia (MD) is a task‐specific neurological movement disorder that impairs performance‐related fine motor control in highly skilled musicians. The symptoms include the deterioration of voluntary control of skilled movement patterns when playing a musical instrument. For pianists, the start of the disorder frequently manifests as a subtle loss of control in fast passages, finger‐curling or involuntary finger extension, irregularity of trills, or fingers appearing to freeze during key release [1, 2]. MD is considered a network disease with reduced inhibition in motor‐related cortical and subcortical regions [3, 4, 5], resulting in reduced inhibition of cortico‐basal‐ganglia‐cerebellar loops [4, 5], reduced surround inhibition [6, 7], and, importantly, hyperactivity of the affected motor cortex [8, 9].

Current treatment options include ergonomic adaptations [10], pharmacological treatments [11] (e.g., anticholinergics and botulinum toxin injections), and behavioral retraining [12]. The combination of botulinum toxin injections and behavioral retraining seems to be most promising; however, achieving sustained motor improvement remains difficult, with a lack of established effective therapies that completely restore motoric abilities in MD [13].

Given the complex pathophysiology of MD and the lack of effective therapies, new therapeutic approaches involving noninvasive transcranial direct current stimulation (tDCS) have gained interest. In tDCS, low electrical current is applied via electrodes on the scalp over specific brain regions to modulate cortical excitability and neural plasticity via long‐term potentiation (LTP) and long‐term depression (LTD). Cathodal stimulation is associated with decreased cerebral excitability via hyperpolarization of neuronal membrane polarity, and anodal stimulation with increased excitability via depolarization [14].

For MD, the restoration of dysfunctional motor cortex activation patterns via tDCS is currently a hypothesis‐guided intervention to improve fine motor control, although several attempts to improve hand dexterity in patients affected by MD with tDCS interventions have yielded mixed results. A recent review [15] described that out of eight tDCS trials, only two randomized controlled trials and three quasi‐experimental studies showed evidence of efficacy. These trials applied tDCS along with motor activation or rehabilitative exercises of finger movements [16, 17].

In a previous controlled, randomized, and double‐blinded study [16], we applied bilateral tDCS over sensory‐motor cortices in 20 pianists (10 with focal dystonia, 10 healthy controls), while they performed bimanual mirrored finger movements. We reported a marked improvement in the temporal accuracy of sequential finger movements with the affected hand during and after stimulation; this was seen in a bi‐hemispheric montage, where the sensorimotor cortex contralateral to the affected hand was inhibited through cathodal stimulation, which causes hyperpolarization. The ipsilateral sensorimotor cortex was facilitated with anodal stimulation. The rationale of bi‐hemispheric intervention lies in the cathodal suppression of the overactive brain region associated with focal dystonia, while anodal stimulation increases the excitability of the normal cerebral motor patterns. In that study, we [16] postulated that this could facilitate interhemispheric crosstalk across the corpus callosum that aids the transfer of healthy motor patterns as an efference copy to the maladaptive cortex that is contralateral to the affected hand. Neither the reverse stimulation—cathodal ipsilateral and anodal contralateral—nor sham stimulation yielded improvements in fine motor control. Furthermore, bi‐hemispheric stimulation without concurrent sensorimotor training failed to improve fine motor control, which emphasizes the importance of combined retraining and stimulation for restoring dystonic symptoms. These stimulation protocols failed to enhance movement accuracy in healthy pianists, a finding that was replicated and confirmed with larger groups of healthy pianists [18]. Although there was considerable improvement in pianists with MD that was more pronounced when dystonia symptoms were more severe, the effects only lasted until 4 days after the stimulation [19].

Following the above‐mentioned stimulation protocol with a bi‐hemispheric montage, Marceglia et al. [20] used tDCS with bilateral focal hand dystonia with two electrodes of the same polarity (i.e., two electrodes with anodal stimulation over both cortices or two electrodes with cathodal stimulation) and a prolonged training protocol of 5 days of stimulation because prolonging stimulation seemed to improve the effect of tDCS. Similarly, Rosset‐Llobet et al. [17] applied tDCS stimulation for 14 days.However, in contrast to our previous study [16], the Rosset‐Llobet study used a complex rehabilitation protocol on unilateral movements that only targeted the affected hand, finding that bilateral cathodal stimulation yielded a subjective improvement of hand dexterity, but only minor effects on motor behavior. The study did not include objective measures of fine motor skills as used in our previous report [16], which is relevant because in MD there is often a discrepancy between subjectively perceived improvements and objectively measured effects on motor performance [21].

The aim of our current study was to evaluate the effects of bi‐hemispheric tDCS: (1) Cathodal stimulation over the sensorimotor cortex contralateral to the affected dystonic hand, and (2) anodal stimulation over the cortex ipsilateral to the affected hand (e.g., if the right hand was affected by dystonia, the cathode was placed on the left motor cortex and the anode on the right motor cortex). This montage was in accordance with our previous study, where the same stimulation protocol was applied during sensorimotor training [16]. In addition, we introduced three important modifications to our original protocol [16] to assess long‐term improvements. First, stimulation occurred on 3 consecutive days instead of every 2 weeks; second, stimulation time was increased from 24 to 30 min. These two changes were incorporated to enhance the cumulative neuromodulatory effects of the stimulation, as repeated tDCS over consecutive days has been shown to enhance motor skill acquisition [22]. Third, video ratings by six expert raters were included to evaluate subtle changes in hand performance with high sensitivity [23]. The temporal accuracy of piano playing was assessed with Musical Instrument Digital Interface (MIDI) analysis. We hypothesized that LTP and sensor‐motor procedural learning would be enhanced by tDCS and the new protocol, and that these effects would be measurable at 10 days after stimulation.

## 2 Materials and Methods

### 2.1 Participants

Ten professional pianists with MD (eight right‐handed) and 10 healthy professional pianists (nine right‐handed) gave informed consent prior to participating in this study. Inclusion criteria for healthy pianists and those with dystonia were musical training with a major in piano and a profession as a pianist (i.e., freelancer, orchestra work, etc.). Participants with focal dystonia had unilateral hand task‐specific focal dystonia. They underwent a full neurological exam by movement disorders specialists (authors E.A. and A.L.) at the outpatient clinic of the Institute of Music Physiology and Musician's Medicine (IMMM) in Hannover. The diagnosis was based on task‐specific, painless, involuntary curling of fingers of the hand and, in some cases, slight extension of the adjacent finger movements and reduced dexterity when playing the piano. No other neurological symptoms were present, and magnetic resonance imaging (MRI) scans at first presentation in the IMMM did not reveal any abnormalities. Exclusion criteria for pianists with dystonia were (1) botulinum toxin A injections up to 3 months prior to the study; (2) bilateral focal dystonia; and for both groups, (3) epilepsy or (4) other neurological diseases or psychiatric diseases (i.e., depression). Demographic data and previous treatments are shown in Tables 1 and 2. Several pianists with MD had previous treatments. The last botulinum toxin injections occurred 3−12 months prior to study entry.

**TABLE 1:** Sample characteristics.

	Pianists with MD (*n* = 10)	Healthy pianists (*n* = 10)	Raters (*n* = 6)	*p*‐value
Gender (male/female)	5/5	5/5	3/3	0.780
Age (years)	44.6 ± 10.8	36.7 ± 12.5	23.0 ± 2.1	0.000
Age range (years)	26−59	26−60	21–26	−
Age, when beginning the instrument (years)	8.5 ± 3.7	6.0 ± 1.6	6.3 ± 3.4	0.138
Handedness (left/right)	2/8	1/9	4/2	0.530
Music genre (Jazz/Classical)	1/9	1/9	0/6	0.732
Highest level of education (*n*)				0.043^a^
Bachelor	1	1	5	
Master/Diploma	4/4	4/4	1	
Other	1	1	−	
Age at onset of MD (years)	34.8 ± 8.2	—	—	—

*Note*: There was an overall difference in education level between groups, but pairwise comparisons showed no significant specific differences between groups.

Abbreviation: MD = musician's dystonia

^a^ Statistical significance was assessed with the Kruskal‐Wallis test.

**TABLE 2:** Characteristics of the pianists with MD.

MD pianists	Gender	Previous treatment	Handedness	Affected digits
1	Female	Anticholinergics, BTX, pedagogical retraining	Right	Index finger
2	Female	BTX, anticholinergics, pedagogical retraining	Right	Middle, fourth, and little finger
3	Male	BTX, anticholinergics	Right	Index finger
4	Female	Benzodiazepin, BTX, SSRI	Right	Index finger
5	Female	BTX, anticholinergics	Right	Index finger
6	Male	BTX, pedagogical retraining	Right	Middle finger
7	Male	No treatment	Right	Middle and index finger
8	Male	Anticholinergics, BTX	Right	Middle finger

Healthy participants were recruited via advertisements, e‐mail, and social media from music schools, universities, and orchestras; pianists with focal dystonia were randomly recruited from the outpatient clinic of the IMMM. Demographic information was obtained via a questionnaire. Participants were matched for gender, age, and handedness. The study protocol was in accordance with the Declaration of Helsinki and approved by the Ethics Committee of the Hanover Medical School.

### 2.2 Experimental Design

**FIGURE 1:**
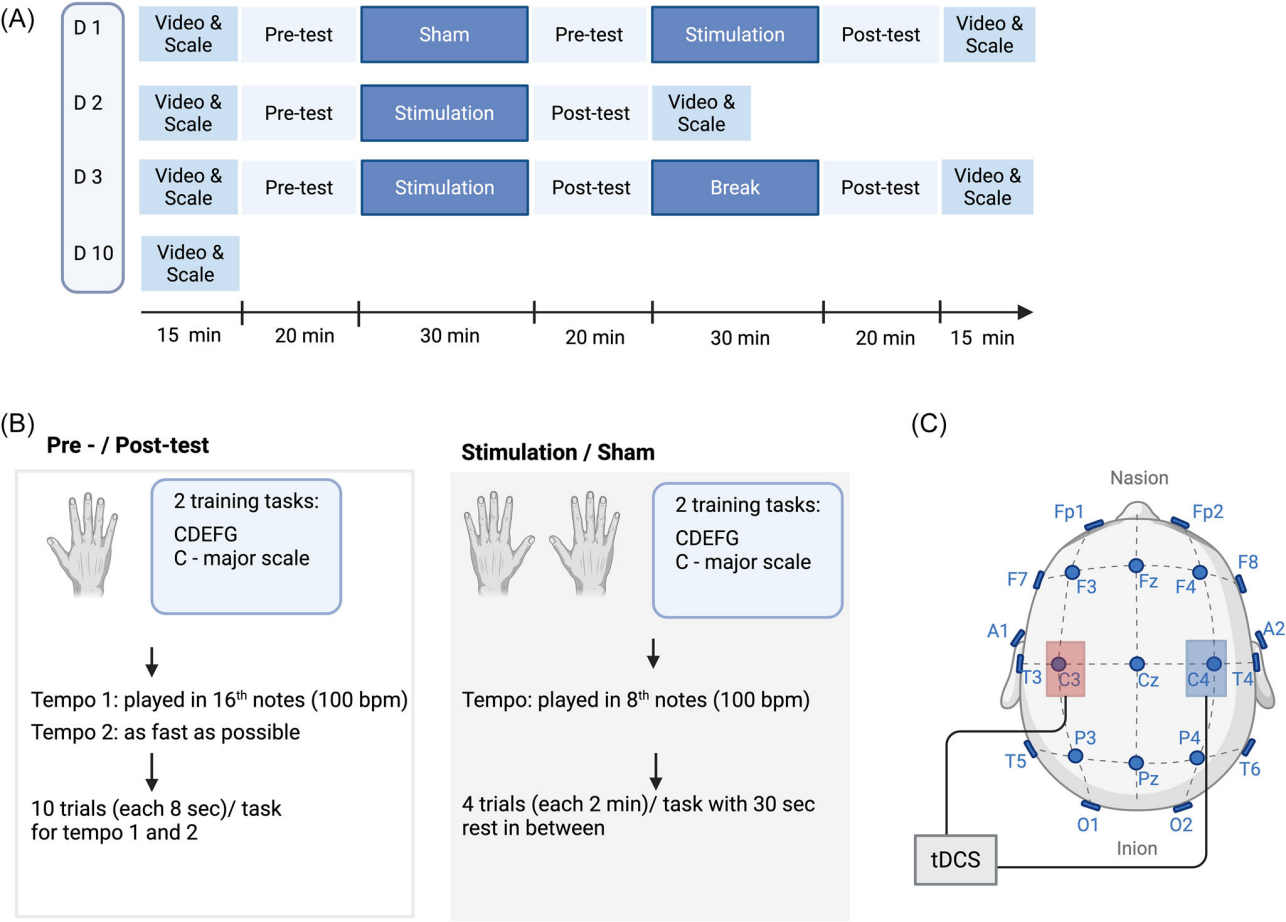
Experimental paradigm and tasks. (A) Time course and organization of the sessions, D = day. (B) Description of experimental tasks that served as hand training. In pre‐ and post‐tests, the affected hand was playing the tasks. In the stimulation condition, both hands were playing the tasks but mirrored (left: CDEFG, right: GFEDC, respectively). (C) Montage of tDCS electrodes according to the 10–20 system. The cathode was placed over the left motor cortex, and the anode over the right motor cortex in right‐handed participants. The montage was reversed in left‐handed participants. The figure was created with biorender.com.

In this case‐control study, subjects participated in three experimental sessions on 3 consecutive days, which were followed by a video measurement 10 days post‐experiment. All participants were assigned to the same experimental conditions, and a double baseline design on day 1 (pretest 1, pretest 2) was employed to discern between possible placebo and training effects. The third day included 30 min of rest between the first posttest (posttest 1) and the second posttest (posttest 2) to evaluate whether any stimulation effects could still be detected after this time period. The detailed protocol is presented in Figure 1.

### 2.3 Objective Assessment of Motor Control

In each pre‐ and posttest, participants played both a repetitive sequential 5‐finger task of adjacent keys with their dominant hand (i.e., right hand C‐D‐E‐F‐G; left hand G‐F‐E‐D‐C) and a C‐major scale spanning two octaves (see Figure 1A). The tasks were played in two tempi: tempo 1 with four 16th notes in synchrony with a metronome set at 100 bpm resulting in an inter‐onset interval (the time lag between two consecutive keystrokes— and the variability being a measure of the evenness of playing [24]) of 150 ms; and tempo 2, the same notes but playing “as fast as possible” (see Figure 1B). During each session, participants played each exercise for 8 s repeatedly 10 times at tempo 1 and then at tempo 2. To avoid fatigue during the 30 min of stimulation, the training tasks were played only in one tempo and at half velocity at 100 bpm (eighth notes with quarter at 100 bpm, inter‐onset interval = 300 ms; see Figure 1B). The 5‐finger‐task was performed four times for 2 min, each with 30 s of rest in between, with a total duration of 10 min. The C major scale was played with the same conditions, for a duration of 10 min. After every playing block of 10 min, a break of 5 min was included to avoid fatigue in participants. Only the 5‐finger task was selected for statistical analysis because of its lower variance compared to the C‐major scale task [24]. The piano was connected to a computer that captured MIDI data using a custom LabVIEW (National Instruments) script.

### 2.4 Transcranial Direct Current Stimulation

After the pretest, participants underwent tDCS stimulation. Two saline (NaCl 0.9%) soaked sponge electrodes (6.5 cm × 5 cm) were placed at C3 and C4 (motor cortex) (see Figure 1C), in accordance with the international 10–20 electroencephalogram system and held in place with a rubber band. Nonsham tDCS (“Stimulation”) was applied via a NeuroConn Stimulator DC (Neurocare, Ilmenau, Germany) with an amplitude of 2 mA (zero‐to‐peak) for 30 min. “Sham” stimulation was applied for 30 s at the beginning of the 30‐min experimental condition. During both Stimulation and Sham conditions, current was ramped up to 2 mA (zero‐to‐peak) over 8 s and then down to 0 mA over 8 s, and tDCS was applied to both hemispheres simultaneously. The motor cortex contralateral to the affected hand received cathodal stimulation (Figure 1C), which is associated with reduced cerebral excitability. The ipsilateral cortex received anodal stimulation, which is associated with increased cerebral excitability. During all measurements, impedance was kept below 10 kΩ.

### 2.5 Rating Scales

#### 2.5.1 Subjective Ratings

At the start and end of each experimental day, participants were asked to rate their playing ability on a visual analogue scale (VAS) [25] ranging from 0% (very poor playing ability) until 100% (very good playing ability).

#### 2.5.2 Video Recordings and Ratings

A video of participants performing two octaves of the C‐major scale was recorded before and after intervention in both participant groups, on days 1–3 and day 10 post‐intervention (retention video). The C‐major scale had to be played with one hand and synchronously to a metronome (100 bpm) as evenly as possible and in 16ths (four notes per beat). In contrast to the hand training task described above, the C‐major scale was analyzed instead of the 5‐finger task because there was no previous literature supporting an advantage of the 5‐finger task in video ratings. Video raters, six master students from piano classes of the university, underwent a 1.5‐h training by authors E.A. and A.L. to learn about typical symptoms of MD in pianists and identify the different levels of severity of dystonia. Raters were instructed on how to mark the VAS.

Under supervision, raters evaluated participant performances on a 15 cm VAS, from 0 to 10, for two items: (1) the temporal rhythmic regularity of the keystrokes (0 = very regular; 10 = very irregular) and (2) motoric impairment during hand movement (0 = no impairment; 10 = strong impairment). The raters assessed the videos in a random order and were blinded to both group affiliation (patients/healthy controls) and experimental condition (pre‐/post‐intervention). Internal consistency of the scale was measured with Cronbach's coefficient alpha, following a previous study reporting the validity of this video rating method in pianists suffering from MD [22].

### 2.6 Statistical Analysis

#### 2.6.1 Motor Performance

Statistical analysis of data were performed with R (version 3.3.2) and SPSS (version 24.0). The time of each keystroke and release (MIDI data) were recorded with a custom‐made LabVIEW (version 8.6) script [26].

Fine motor control was assessed by measuring the temporal variability of the key stroke and key releasing intervals. This was calculated as the standard deviation of the inter‐keystroke interval (IKI‐SD), which is the interval between two consecutive key strokes and the inter‐key‐release interval (IRI‐SD), the interval between two consecutive key releases. Lower values of IKI‐SD and IRI‐SD suggested a good playing performance with low rhythmic variability and high temporal precision. Two participants (two left‐handed) with focal dystonia and two healthy controls (one right‐handed, one left‐handed) had to be removed from statistical analysis due to technical problems with the MIDI measurements. Therefore, only eight subjects in each group (all were right‐handed) entered into the final analysis.

Since the data did not meet the assumptions of normality, the Friedman test was employed to assess differences in temporal playing variability of keystrokes (IKI‐SD) and key releases (IRI‐SD) across conditions per group. This was done for the factors Condition (2 levels: pretest, posttest) and Day (3 levels: 1–3). The Wilcoxon signed‐rank test with Bonferroni correction was used for post‐hoc analysis.

Next, the Mann–Whitney *U* test was used to delineate differences in temporal playing variability between patients and healthy controls. Further, in order to discern possible placebo effects on the first day from carry over effects on the third day, the Wilcoxon signed‐rank test was used, whereby the two pretests on day 1 (pretest 1, pretest 2) and the two posttests on day 3 (posttest 1, posttest 2) were each compared for IRI‐SD and IKI‐SD, respectively (see Figure 1A for experiment protocol). The effect size *r* was calculated, with values of 0.1 until 0.3 to be considered small effects, from 0.3 to 0.5 to be moderate, and from 0.5 onward to be large effects [27].

#### 2.6.2 Subjective Rating Analysis

We collected 140 subjective ratings. Thirty‐five ratings were removed due to missing data from five participants (four patients, one healthy control). The data of the remaining 15 participants consisted of 105 subjective ratings and failed to meet the assumptions of normality (based on the Shapiro−Wilk test); therefore, a Friedman test was used. Subjective ratings were analyzed separately from analyzed video ratings, since we did not expect an interaction effect of video ratings and subjective ratings. The Friedman test was employed per group for Condition (2 levels: pretest, posttest) and Day (4 levels: days 1, 2, 3, and 10). Between‐group differences in subjective ratings were tested with the Mann−Whitney *U* test for all conditions and all time points. Wilcoxon signed‐rank tests were used for post‐hoc analysis, and the effect size Cohen's *r* was calculated [27]. It was not possible to compute the intraclass correlation coefficient (ICC) for the subjective ratings due to the design of the scale.

#### 2.6.3 Video Rating Analysis

A total of 140 videos of hand performances were recorded, and 28 videos were excluded due to incorrect measurements (two patients, two controls). Additionally, 14 videos were excluded from participants with missing videos (two patients). Missing videos resulted either from technical recording problems or incompletely filled‐in ratings. The remaining 98 videos of six patients and eight healthy controls were used for analysis

All videos were randomly allocated to six raters, and each video was assessed by two different raters. Further, each video was rated twice, which resulted in four ratings per video. The total of 98 included videos amounted to 392 ratings.

In the analysis of hand performance, the average of two individual ratings for one video per condition was computed to compare ratings between conditions. Thereafter, explorative data analysis revealed two outliers (>3× IQR over the 0.75 quartile) from one patient. For each of these two values, the mean from the remaining six ratings of patients was calculated and used as a replacement in further analysis due to the small sample size. As the data were normally distributed (based on the Shapiro−Wilk test), a repeated measures ANOVA (RM ANOVA) with Group as a between‐subjects factor, and Condition and Day as within‐subjects factor, was employed; the remaining assumptions for usage of this test were fulfilled.

Further, an ICC was calculated to assess inter‐rater reliability. We used a two‐way random ICC, which measures absolute agreement, whereby the systematic differences between raters were important. Values of ICC range between 0 and 1, with 0.40 being poor reliability, between 0.40 and 0.59 being fair, values from 0.60 being good, and values above 0.75 being considered as excellent rater reliability [28]. The effect size $\eta_{p}^{2}$ was calculated to assess the magnitude of effects.

## 3 Results

### 3.1 Demographics

There was a significant difference in age between groups according to the Kruskal−Wallis test (*H*(2) = 14.29, *p_adj_* < 0.001, ε^2^ = 0.57). Pairwise comparisons with Dunn's procedure and Bonferroni correction revealed that raters (*Mdn*. = 22.50) were significantly younger than both patients ([*Mdn*. = 45.50], *p_adj_* = 0.001) and healthy controls ([*Mdn*. = 30.00], *p_adj_* = 0.02) (comparisons, see Table S1.1 and Figure S1.1). There was also an overall difference in education level between groups (*H*(2) = 6.30, *p_adj_* = 0.04, ε^2^ = 0.25); however, pairwise comparisons revealed no significant differences after adjusting for multiple comparisons (see Table S1.2 and Figure S1.2). Most participants reported no adverse events, although one experienced a slight scalp burn that healed without complications within days after the last session.

### 3.2 Motor Performance

**FIGURE 2:**
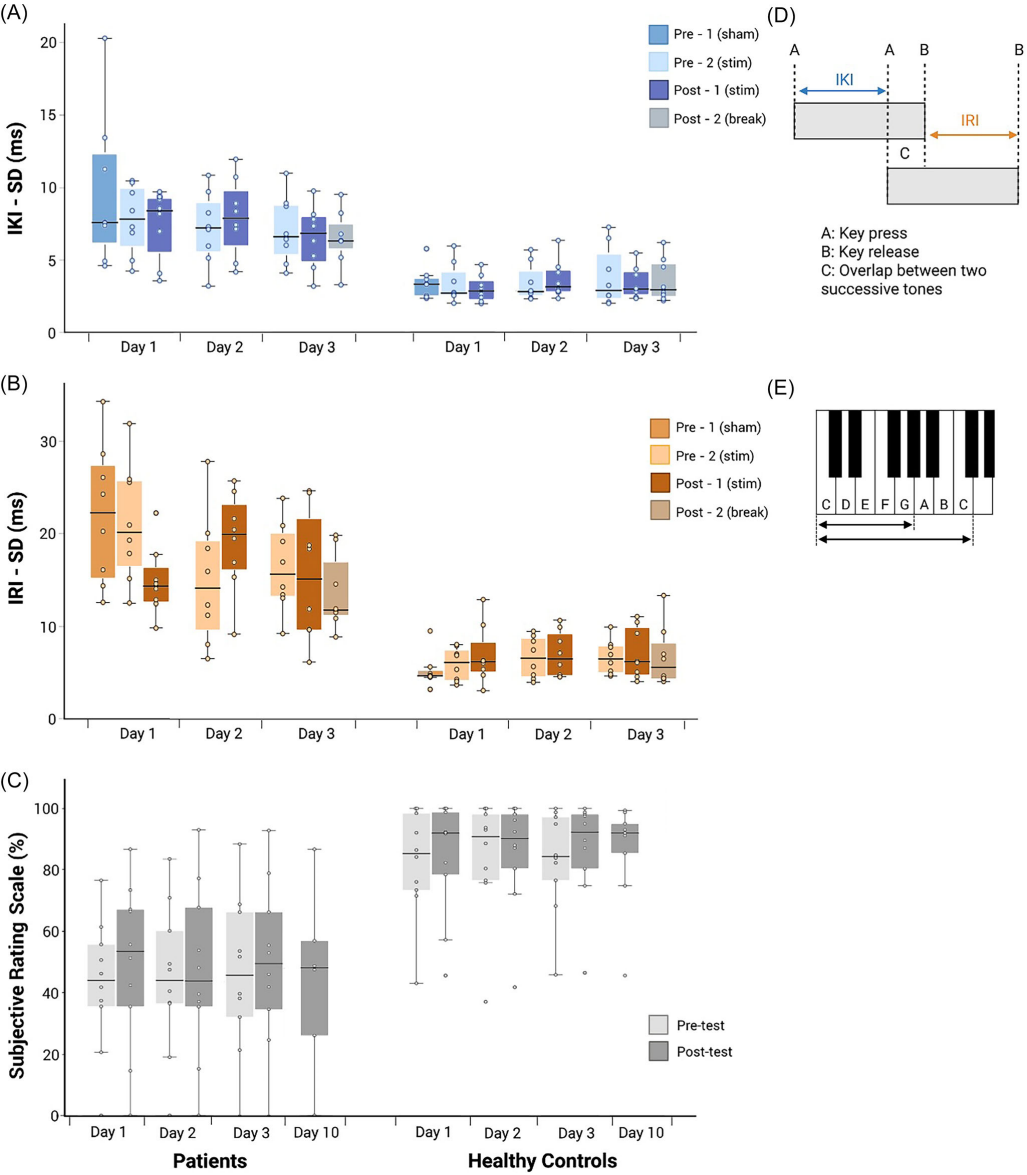
Boxplots for inter‐keystroke‐intervals (IKI‐SD) and inter‐keyrelease‐intervals (IRI‐SD) and subjective ratings. Individual data points of inter‐keystroke‐intervals (IKI‐SD) and inter‐keyrelease‐intervals (IRI‐SD) and subjective ratings are shown as dots. (A) The variability of time intervals between keypresses, IKI‐SD, and (B) between key releases, IRI‐SD, which were both played in the playing condition “as fast as possible.” Lower values of IRI‐SD and IKI‐SD indicated a good playing ability with low rhythmic variability and high temporal precision. (C) Subjective ratings of patients and controls. Pre‐ and post‐test refer to the time points at the beginning and end of the experimental day (days 1–3, and one measurement on day 10). Higher percentages indicate better subjective performance ability in the exercises. (D) Diagram explaining the variables of IKI‐SD and IRI‐SD. (E) Schematic keyboard explaining the different hand tasks: (1) CDEFG (5‐finger‐task), (2) C‐major scale.

The medians and interquartile ranges of IKI‐SD and IRI‐SD are given in Figure 2A,B and Table 3. Regarding the assessment of placebo or carryover effects, the Wilcoxon signed‐rank test with Bonferroni correction (α = 0.05/8 = 0.006) revealed no significant differences between pretest 1 and 2 in performance for the sham condition on day 1 and no carryover effects of tDCS between posttest 1 and 2 after 30 min for either patients or healthy controls on day 3.

**TABLE 3:** Pairwise comparisons of the MIDI data.

Group	Pair			Median (interquartile range)		*Z*	*p*‐value
Patients	IKI‐SD	Pre‐1	/Pre‐2	7.58 (5.53−2.90)	/4.23 (5.44−10.16)	−1.68	0.09
(*n* = 8)		Post‐1	/Post‐2	6.81 (4.66−8.04)	/6.31 (5.44−7.86)	−0.70	0.48
	IRI‐SD	Pre‐1	/Pre‐2	22.25 (14.77−27.97)	/20.10 (15.80−25.78)	−0.98	0.33
		Post‐1	/Post‐2	15.01 (9.56−22.91)	/11.66 (10.93−18.08)	−0.42	0.67
Controls	IKI‐SD	Pre‐1	/Pre‐2	3.31 (2.45−3.84)	/2.71 (2.54−5.33)	−0.42	0.67
(*n* = 8)		Post‐1	/Post‐2	2.96 (2.51−5.08)	/2.92 (2.37−5.77)	−0.98	0.33
	IRI‐SD	Pre‐1	/Pre‐2	4.62 (4.50−5.42)	/6.09 (4.09−7.67)	−1.31	0.23
		Post‐1	/Post‐2	6.07 (4.59−10.06)	/5.48 (4.26−8.71)	−0.70	0.48

The Friedman test showed that pianists with MD displayed significant differences in the overall temporal variability of key releases between sham and stimulation conditions (IRI‐SD: *χ* ^2^(7) = 25.17, *p* < 0.001, *W* = 0.45), but not in keystrokes (IKI‐SD: *χ* ^2^(7) = 6.17, *p* = 0.52, *W* = 0.11). Healthy controls showed no differences in the temporal variability of keystrokes and key releases after sham or stimulation conditions (IKI‐SD: *χ* ^2^(7) = 7.25, *p* = 0.40, *W* = 0.13; IRI‐SD: *χ* ^2^(7) = 13.38, *p* = 0.06, *W* = 0.24).

Post‐hoc analysis with Wilcoxon signed‐rank tests corrected with Bonferroni (α = 0.05/7 = 0.007) revealed no significant differences between sham and stimulation conditions for patients regarding the temporal variability of key releases. In MD patients, there were no significant differences between sham and stimulation conditions on key releases on the first day, between pretest 1 and posttest 1 (*Z* = –2.52, *p* = 0.01, *r* = 0.89) and between pretest 1 on the first day and pretest on the second day of the intervention (*Z* = –2.52, *p* = 0.01, *r* = 0.89). Further, there was no significant difference between conditions on key releases between pretest 1 on the first day and posttest 2 on the third day (*Z* = –2.52, *p* = 0.01, *r* = 0.89).

The Mann–Whitney *U* test corrected with Bonferroni for multiple comparisons revealed a significant difference (α = 0.05/8 = 0.006) in motor performance (measured as temporal variability) between patients and healthy controls in all conditions for keystrokes and key releases:

### 3.3 Keystrokes

- Day 1: pretest 1, *U* = 2.00 (*Z* = –3.15), *p* = 0.002, *r* = 0.78; pretest 2, *U* = 3.00 (*Z* = –3.04), *p* = 0.002, *r* = 0.75; posttest 1, *U* = 3.00 (*Z* = –3.04), *p* = 0.002, *r* = 0.75;
- Day 2: pretest 2, *U* = 4.00 (*Z* = –2.94), *p* = 0.003, *r* = 0.73; posttest 1, *U* = 3.00 (*Z* = –3.04), *p* = 0.002, *r* = 0.76;
- Day 3: pretest 2, *U* = 10.00 (*Z* = –2.31), *p* = 0.021, *r* = 0.57; posttest 1, *U* = 5.00 (*Z* = –2.83), *p* = 0.005, *r* = 0.71; posttest 2, *U* = 4.00 (*Z* = –2.94), *p* = 0.003, *r* = 0.74).

### 3.4 Key Releases

- Day 1: pretest 1, *U* = 0.00 (*Z* = −3.36), *p* = 0.001, *r* = 0.84, pretest 2, *U* = 0.00 (*Z* = –3.36), *p* < 0.001, *r* = 0.84; posttest 1, *U* = 4.00 (*Z* = −2.94), *p =* 0.003, *r =* 0.73;
- Day 2: pretest 2, *U* = 7.00 (*Z* = –2.62), *p* = 0.009, *r* = 0.65; posttest 1, *U =* 2.00 (*Z* = –3.15), *p =* 0.002, *r =* 0.78;
- Day 3: pretest 2, *U* = 1.00 (*Z = –*3.25), *p =* 0.001, *r =* 0.81; posttest 1, *U* = 8.00 (*Z* = –2.52), *p =* 0.01, *r =* 0.63; posttest 2, *U* = 6.00 (*Z* = –2.73), *p =* 0.006, *r =* 0.68).

To summarize, statistical analysis of motor performance found that evenness of keystroke and key releases in healthy pianists was higher than in pianists suffering from dystonia. Data points for individual pianists can be found in Figure S3.

### 3.5 Subjective Ratings

The Friedman test showed a significant overall difference in subjective ratings across conditions in the pianist dystonia group (*χ* ^2^(6) = 12.95, *p* = 0.04, *W* = 0.27). Differences among healthy pianists in self‐ratings of hand performance across conditions were not significant (*χ* ^2^(6) = 3.28, *p* = 0.77, *W* = 0.07). A post‐hoc analysis with pairwise comparisons (in SPSS) with Bonferroni correction for multiple comparisons showed no significant difference in subjective hand performance across conditions (statistical significance with multiple comparisons was set at *p* = 0.002) (details can be seen in Table S2). In addition, subjectively rated playing ability 10 days after stimulation was not significantly different from playing ability rated on the first day before intervention in both MD patients (*Z* = –1.75, *p* = 0.08, *r* = 0.46) and controls (*Z* = –0.56, *p* = 0.58, *r* = 0.14).

However, the Mann–Whitney *U* test with Bonferroni correction (α = 0.05/7 = 0.007) revealed significant differences in subjective ratings between groups across almost all conditions:

Day 1: pretest, *U* = 8.00 (*Z* = –3.18), *p* = 0.001, *r* = 0.79; posttest, *U* = 12.00 (*Z* = –2.87), *p* = 0.004, *r* = 0.72;
Day 2: pretest, *U* = 9.00 (*Z* = –3.10), *p* = 0.002, *r* = 0.77; posttest, *U* = 11.00 (*Z* = –3.02), *p* = 0.002, *r* = 0.75;
Day 3: pretest, *U* = 11.00 (*Z* = –2.95), *p* = 0.003, *r* = 0.74; posttest, *U* = 10.00 (*Z* = –2.83), *p* = 0.002, *r* = 0.70;
Day 10: posttest, *U* = 6.00 (*Z* = –2.45), *p* = 0.013, *r* = 0.61.

In summary, healthy controls rated their subjective playing ability significantly better than did MD patients (see Figure 2 for medians and interquartile ranges (IQR).

### 3.6 Video Ratings

**FIGURE 3:**
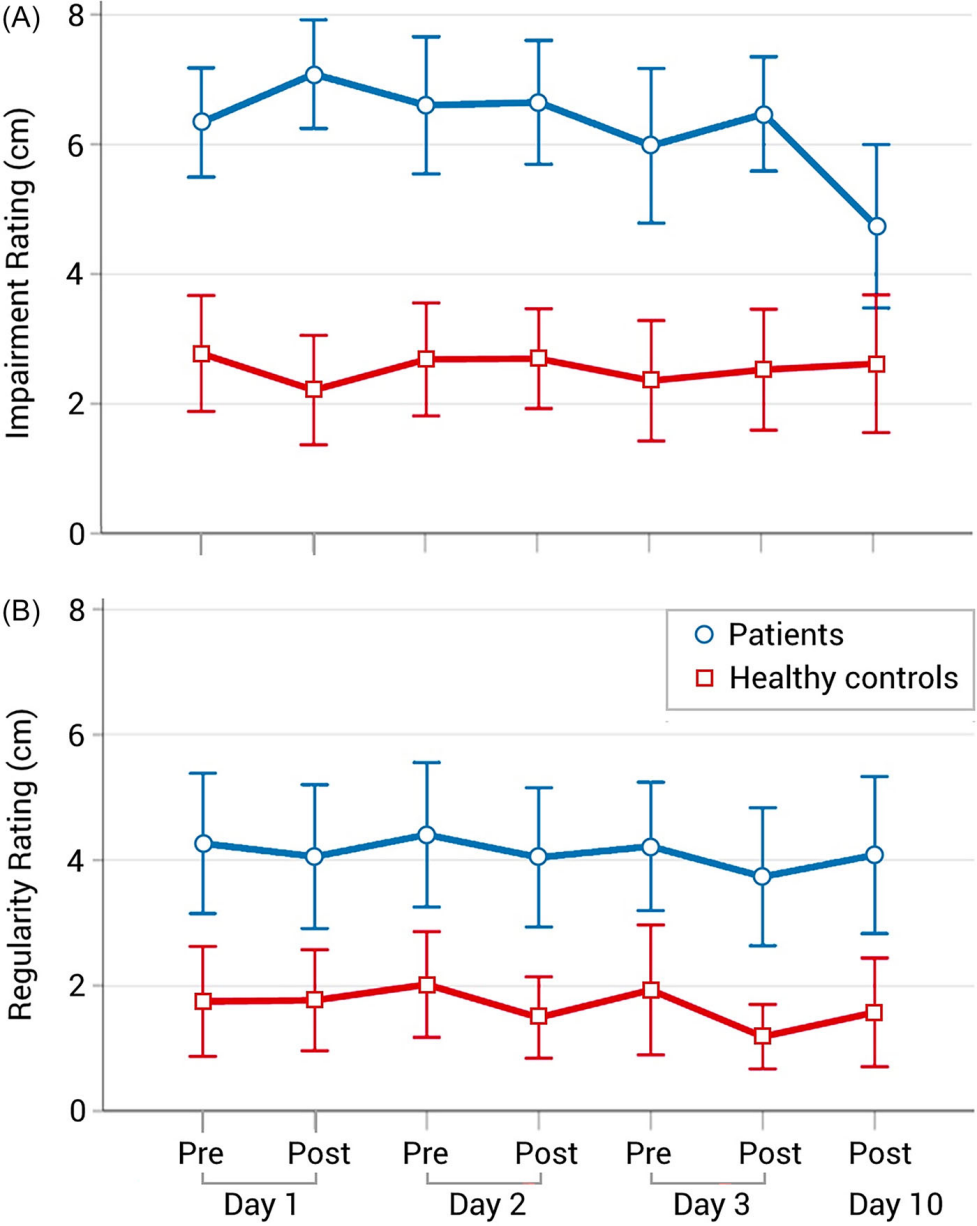
Video ratings. (A) Mean scores for video ratings on the items movement impairment and (B) regularity. Error bars represent the standard error of the mean. Videos were rated on a VAS scale. Higher values in A indicate a higher movement impairment, whereas higher values in B indicate better playing regularity.

Descriptive statistics of video ratings are presented in Figure 3. The two items of the video rating scales were analyzed separately.

A two‐way random ICC showed a poor inter‐rater reliability for the scale item *regularity* (ICC = 0.04, *F*(118, 595) = 1.3, *p* = 0.04, 95% CI [0.04, –0.03]). In contrast, *movement impairment* showed a good inter‐rater reliability (ICC = 0.70, *F*(118, 595) = 3.4, *p* < 0.01, 95% CI [0.61, 0.78]).

An RM ANOVA revealed no total significant differences in ratings of regularity or movement impairment between the sham and stimulation conditions (regularity: *F*(1, 12) = 2.90, *p* = 0.11, $\eta_{p}^{2}$ = 0.20; movement impairment: *F* (1, 12) = 0.003, *p* = 0.96, $\eta_{p}^{2}$ = 0.00). And there were no significant interaction effects: Condition × Group [regularity: *F*(1, 12) = 0.41, *p* = 0.54, $\eta_{p}^{2}$ = 0.03, movement impairment: *F*(1, 12) = 1.7, *p* = 0.22, $\eta_{p}^{2}$ = 0.12]; Day × Group [regularity: *F*(2, 24) = 1.13, *p* = 0.34, $\eta_{p}^{2}$ = 0.09, movement impairment: *F*(2, 24) = 0.60, *p* = 0.56, $\eta_{p}^{2}$ = 0.05]; Condition × Day [regularity: *F*(2, 24) = 1.99, *p* = 0.16, $\eta_{p}^{2}$ = 0.14; movement impairment: *F*(2, 24) = 0.22, *p* = 0.80, $\eta_{p}^{2}$ = .02]; and Condition × Day × Group (regularity: *F*(2, 24) = 0.19, *p* = 0.83, $\eta_{p}^{2}$ = 0.02; movement impairment: *F*(2, 24) = 0.07, *p* = 0.93, $\eta_{p}^{2}$ = 0.01). However, there was a significant difference between healthy and MD groups for regularity and movement impairment (regularity: *F*(1, 12) = 22.97, *p* < 0.001, $\eta_{p}^{2}$ = 0.66, movement impairment: *F*(1, 12) = 29.64, *p* < 0.001, $\eta_{p}^{2}$ = 0.71).

## 4 Discussion

The treatment of MD remains challenging because of its complex pathophysiology as a network disorder. The inhibitory and excitatory interactions between sensorimotor, limbic, and executive brain circuits require treatment options with new approaches. Our study here investigated the effects of 3 days of bi‐hemispheric tDCS with simultaneous sensory‐motor training on fine motor control in MD pianists. Further, we examined whether there was a sustained effect of tDCS on fine motor control 10 days after intervention. We also investigated whether a controlled video‐rating procedure could serve as an additional objective measurement in MD. While our results, by both video and subjective ratings, showed no effect of tDCS on fine motor control and no effects 10 days post‐intervention, we observed a pre‐test deficit in fine motor control in patients compared to healthy control pianists, measured as differences in the playing variability of inter‐key‐release intervals (IRI) and inter‐keystroke intervals (IKI). These results differ from our previous study [16].

Even though we found no effect of tDCS, we observed fluctuations in IRI and IKI in MD patients: There was a nonstatistically significant trend toward improved temporal variability at the end of the session (e.g., see post‐test 1 in Figure 2) between the second and third day of the experiment. However, the data were variable and increased in variability after the first day.

Compared to the study of Furuya et al. [16], our results could be different because of our specific protocol and choice/size of subject population. As to the former, the motor task was executed in two tempi: In tempo 1, 16th notes (four notes per beat) were played synchronously to a metronome with 100 bpm; in tempo 2, participants played as fast as possible. We analyzed only tempo 2 because it provides a good discrimination between patients and healthy subjects and could provoke symptoms of dystonia in patients. However, the fast speed of tempo 2 reportedly can lead to muscular fatigue not only in patients with MD but also in healthy controls [29].

Of particular relevance to our study, muscular fatigue can induce deterioration of motor control due to peripheral and central neurophysiological mechanisms and oppose potential effects of the tDCS intervention [30]. The effects of fatigue on dystonic symptoms can vary greatly. Pesenti et al. [31] showed short‐term beneficial effects of muscular fatigue in patients suffering from musician's cramp; however, the improvement lasted less than 5 min after the fatiguing task ended. Because performance in the retention test occurred after 10 days, the timeframe of the observed muscle fatigue by Presenti et al. is not likely relevant in our study. In the study of Furuya et al. [16], patients and controls participated in a total of five sessions (with four stimulation protocols), each separated by more than 2 weeks, and the participants were stimulated for 24 min per session. Consequently, the total duration of each intervention day in the current study was longer (approximately 1.6 h per day) and hence had a higher potential for causing muscular fatigue. The sample size in Furuya et al. [16] was 20 participants (10 MD, 10 controls), thereby slightly increasing the chance to detect an effect. They had fewer multiple affected fingers, a slightly lower average age, and fewer patients had received prior botulinum toxin injections, which could also explain the differences in our results.

The questions remain whether an intervention of 3 consecutive days is sufficient to produce an observable effect and whether tDCS intervention with an interruption of several weeks between sessions can improve fine motor control. Given the current results, it may be possible that 3 days of intervention is not long enough to induce changes in hand dexterity and long‐lasting effects; for example, a study by Rosset Llobet et al. [17] with stimulation over 2 weeks showed a subjective improvement of dystonia symptoms. Indeed, motor skill learning is commonly achieved by training over a longer period than a few days, whereas adaptations of motor skills can already occur over a shorter period [32]. Taking this into consideration, we propose that tDCS protocols should last longer than 3 consecutive days and involve stimulation over more than one session.

With respect to the third aim of the study, the video‐rating method showed mixed results. Based on auditory information, raters produced plausible results; however, interrater consistency for the item regularity was low, probably due to different assessments of evenness in playing the sequential hand task. An introductory training session with more in‐depth practice of the criteria for assessing evenness in playing piano could improve the interrater consistency. In contrast, ratings for visually observed hand movement abnormality showed good interrater consistency. As anticipated, the abnormal finger flexions and extensions of dystonia were more salient and easier to detect than the temporal accuracy [22].

The results of the current study are in line with an increasing body of literature that fails to demonstrate efficacy of tDCS in motor disorders. In 2017, a “consensus and critical position paper” presented evidence‐based guidelines for tDCS usage for different conditions, varying from psychological to motor disorders [33]. While tDCS interventions seemed to be efficient in pain and psychiatric disorders, because of predominantly nonreplicated small sample sizes, the studies could not recommend tDCS in focal dystonia; the same was true for tDCS in stroke rehabilitation. In another, more recent review consisting of 19 studies, anodal stimulation yielded small but significant effects of tDCS on motor rehabilitation in stroke patients [34]. Our results here are consistent with the systematic review by Menon et al. [15] examining the effects of repetitive Transcranial Magnetic Stimulation (TMS) and tDCS in focal hand dystonia, which found generally inconsistent and modest results regarding the efficacy of the procedures. Studies employing a larger number of sessions, as well as a combination of tDCS and neurorehabilitative training, showed greater therapeutic effects than tDCS alone. Overall, the current literature suggests that the effectiveness of tDCS in focal dystonia remains inconsistent. Future studies are needed to clarify and provide a better understanding of the potential of tDCS in focal dystonia and other disorders.

### 4.1 Limitations

Considering the small sample size (*n* = 16 for motor assessment, *n* = 14 for video ratings), the results can only be cautiously transferred to the general population of MD patients. Consistent with the previous study by Furuya et al. [16], we aimed at investigating a relatively homogeneous sample of patients and, therefore, focused on pianists. Regarding the motor assessment, the initial sample size was comparable to that of our previous study; however, the exclusion of four participants further limited the statistical power.

Furthermore, recruitment of participants was restricted by the low prevalence of MD, making it difficult to find eligible participants, since it is a rare disorder. Participation in our study was time‐consuming, with sessions over 3 consecutive days and a retention test 10 days later that necessitated an additional visit. These logistical challenges, combined with the rarity of MD, resulted in a relatively small sample size. Also, the nonrandomized allocation of participants, owing to the experimental conditions, could have led to bias and necessitates future replications taking this into consideration. We believe that the double baseline procedure at least ruled out the most relevant placebo effects.

Last, we did not include measurements of cerebral excitability induced by tDCS. Assessing cerebral excitability before and after tDCS intervention by TMS could verify the level of excitatory and inhibitory changes at the motor cortex. For instance, Nitsche and Paulus [35] showed that a longer anodal tDCS of 13 min with 1 mA led to TMS‐measured excitability changes for up to 90 min at the end of the stimulation. Furthermore, Santarnecchi et al. [36] showed that excitability changes in the motor cortex M1 became only evident at the end of tDCS intervention rather than during stimulation, although aftereffects exceeding 30 min were not recorded. However, these studies utilized uni‐hemispheric tDCS interventions and, therefore, are not directly comparable to our design. Future studies should additionally assess cortical excitability by means of TMS in bi‐hemispheric stimulation protocols.

## 5 Conclusion

Three days of bi‐hemispheric tDCS over sensorimotor cortices along with bimanual training did not improve fine motor control in MD immediately after stimulation and after 10 days of retention in a small sample of eight subjects; however, in individual cases, some improvement was observed. Future studies should include larger samples and strive to determine which patients may benefit from tDCS by assessing cortical excitability via TMS before training.

## Author Contributions

Isabella Ampomah conceived the design of the study, recruited healthy subjects, carried out measurements and collected the data, carried out the statistical analysis, and drafted the manuscript. Michael Großbach performed statistical analysis, reviewed and edited the manuscript. André Lee recruited and examined dystonia patients and edited the manuscript. Eckart Altenmüller conceived the design of the study, examined the patients, acquired the healthy probands and participated in data collection and data evaluation, and drafted the manuscript. All authors read and approved the manuscript.

## Funding

This research was funded by the University of Music, Drama and Media Hannover (HMTM). The HMTM provided funding for remunerating travel costs of the subjects and patients and compensation for the time spent during the experiments.

## Conflicts of Interest

The authors declare that they have no conflicts of interest.

## Supporting information




**Supplementary Materials**: nyas70375‐sup‐0001‐SuppMat.docx
