# Supplementary material for: Three‐Day Bi‐Hemispheric tDCS With Motor Training Shows No Improvement in Pianist's With Musician's Dystonia: A Pilot Study
Source: Ann N Y Acad Sci. 2026 Aug 20;1562(1):e70375. doi: 10.1111/nyas.70375 (PMC13492228; doi:10.1111/nyas.70375)
Supplement: Supplementary file 1 [file NYAS-1562-0-s001.docx]

**Supplementary Material**

**Three-Day Bi-Hemispheric tDCS With Sensorimotor Training Shows No Detectable Improvement in Pianists With Musician’s Dystonia: A Pilot Study**

Isabella Ampomah, Michael Großbach, Andre Lee, Eckart Altenmüller

**A. DEMOGRAPHICS**

There were significant differences in age and education level between groups according to the Kruskal-Wallis test (see manuscript). Post-hoc pairwise comparisons were run with SPSS (Dunn´s procedure) for age and education level respectively.

**Table S1. Post-hoc pairwise comparisons between groups for the variable age.**

| Comparison | Test Statistic | Std. Error | Std. Test Statistic (*Z*) | *p*-value | Adj. *p*-value.^a^ |
| --- | --- | --- | --- | --- | --- |
| Raters-Healthy Controls | 10.550 | 3.940 | 2.678 | 0.007 | 0.022 |
| Raters-MD | 14.800 | 3.940 | 3.756 | <0.001 | 0.001 |
| Healthy Controls-MD | 4.250 | 3.412 | 1.245 | .213 | 0.639 |
| Note. Asymptotic significances (2-sided tests) are displayed.  ^a^ Significance values have been adjusted by the Bonferroni correction for multiple tests. | | | | | |

**Figure S1 Boxplots of group comparisons for age in the Kruskal-Wallis test**

**********************

**Table S2. Post-hoc pairwise comparisons between groups for the variable education.**

| Comparison | Test Statistic | Std. Error | Std. test statistic (Z) | *p*-value | Adj. *p*-value.^a^ |
| --- | --- | --- | --- | --- | --- |
| Raters-Healthy Controls | 8,200 | 3,742 | 2,192 | 0,028 | 0,085 |
| Raters-MD | 8,700 | 3,742 | 2,325 | 0,020 | 0,060 |
| Healthy Controls-MD | 0,500 | 3,240 | 0,154 | 0,877 | 1,000 |

Note. Asymptotic significances (2-sided tests) are displayed.

^a^ Adjusted significance values have been adjusted by the Bonferroni correction for multiple tests.

**Figure S2. Boxplots of group comparisons for education in the Kruskal-Wallis test**

**B. SUBJECTIVE RATINGS**

The Friedman`s test was used to analyse differences in subjective ratings. There was a significant difference in ratings in MD patients, post-hoc pairwise comparisons in MD patients were computed with the inbuilt SPSS procedure (using Dunn´s procedure pairwise comparisons).

**Table S3. Post-hoc pairwise comparisons and adjusted significance values for subjective ratings in MD patients.**

| Comparing conditions | Test Statistic (W) | Std. Error | Std. test statistic (Z) | *p*-value | Adj.  *p*-value.^a^ |
| --- | --- | --- | --- | --- | --- |
| D2_pre - D1_pre | 0,250 | 1,247 | 0,200 | 0,841 | 1,000 |
| D2_pre - D2_post | -0,583 | 1,247 | -0,468 | 0,640 | 1,000 |
| D2_pre - D1_post | 1,000 | 1,247 | 0,802 | 0,423 | 1,000 |
| D2_pre - D3_pre | -2,000 | 1,247 | -1,604 | 0,109 | 1,000 |
| D2_pre - D10_post | -2,417 | 1,247 | -1,938 | 0,053 | 1,000 |
| D2_pre - D3_post | -3,083 | 1,247 | -2,472 | 0,013 | 0,282 |
| D1_pre - D2_post | -0,333 | 1,247 | -0,267 | 0,789 | 1,000 |
| D1_pre - D1_post | -0,750 | 1,247 | -0,601 | 0,548 | 1,000 |
| D1_pre - D3_pre | -1,750 | 1,247 | -1,403 | 0,161 | 1,000 |
| D1_pre - D10_post | -2,167 | 1,247 | -1,737 | 0,082 | 1,000 |
| D1_pre - D3_post | -2,833 | 1,247 | -2,272 | 0,023 | 0,485 |
| D2_post - D1_post | 0,417 | 1,247 | 0,334 | 0,738 | 1,000 |
| D2_post - D3_pre | -1,417 | 1,247 | -1,136 | 0,256 | 1,000 |
| D2_post - D10_post | -1,833 | 1,247 | -1,470 | 0,142 | 1,000 |
| D2_post - D3_post | -2,500 | 1,247 | -2,004 | 0,045 | 0,945 |
| D1_post - D3_pre | -1,000 | 1,247 | -0,802 | 0,423 | 1,000 |
| D1_post - D10_post | -1,417 | 1,247 | -1,136 | 0,256 | 1,000 |
| D1_post - D3_post | -2,083 | 1,247 | -1,670 | 0,095 | 1,000 |
| D3_pre - D10_post | -0,417 | 1,247 | -0,334 | 0,738 | 1,000 |
| D3_pre - D3_post | -1,083 | 1,247 | -0,869 | 0,385 | 1,000 |
| D10_post - D3_post | 0,667 | 1,247 | 0,535 | 0,593 | 1,000 |

Note. Asymptotic significances (2-sided tests) are displayed.

^a^ Adjusted significance values have been adjusted by the Bonferroni correction for multiple tests

**Figure S3.** Boxplots for inter-keystroke-intervals (Stdev IKI) and inter-keyrelease-intervals (Stdev IRI) and subjective ratings. Individual data points of participants were linked across different conditions for easier assessment of individual changes of the intervention. (A) The variability of time intervals between keypresses and (B) between key releases in the playing condition “as fast as possible”. (C) Subjective ratings. Lower values of IRI- and IKI-SD indicated a good playing ability with low rhythmic variability and high temporal precision.
